# Neuron-macrophage crosstalk in the intestine: a “microglia” perspective

**DOI:** 10.3389/fncel.2015.00403

**Published:** 2015-10-08

**Authors:** Simon Verheijden, Sebastiaan De Schepper, Guy E. Boeckxstaens

**Affiliations:** Laboratory of Neuroimmune Interaction and Mucosal Immunology, Department of Clinical and Experimental Medicine, Translational Research Center for Gastrointestinal Disorders (TARGID), University Hospital LeuvenKU Leuven, Leuven, Belgium

**Keywords:** neuroimmune, intestinal macrophage, microglia, CX3CR1, enteric nervous system, transforming growth factor β

## Abstract

Intestinal macrophages are strategically located in different layers of the intestine, including the mucosa, submucosa and muscularis externa, where they perform complex tasks to maintain intestinal homeostasis. As the gastrointestinal tract is continuously challenged by foreign antigens, macrophage activation should be tightly controlled to prevent chronic inflammation and tissue damage. Unraveling the precise cellular and molecular mechanisms underlying the tissue-specific control of macrophage activation is crucial to get more insight into intestinal immune regulation. Two recent reports provide unanticipated evidence that the enteric nervous system (ENS) acts as a critical regulator of macrophage function in the myenteric plexus. Both studies clearly illustrate that enteric neurons reciprocally interact with intestinal macrophages and are actively involved in shaping their phenotype. This concept has striking parallels with the central nervous system (CNS), where neuronal signals maintain microglia, the resident macrophages of the CNS, in a quiescent, anti-inflammatory state. This inevitably evokes the perception that the ENS and CNS share mechanisms of neuroimmune interaction. In line, intestinal macrophages, both in the muscularis externa and (sub)mucosa, express high levels of CX3CR1, a feature that was once believed to be unique for microglia. CX3CR1 is the sole receptor of fractalkine (CX3CL1), a factor mainly produced by neurons in the CNS to facilitate neuron-microglia communication. The striking parallels between resident macrophages of the brain and intestine might provide a promising new line of thought to get more insight into cellular and molecular mechanisms controlling macrophage activation in the gut.

## Neuromodulation of Intestinal Macrophages

The enteric nervous system (ENS) acts as a vital regulator of many physiological functions of the gastrointestinal tract including motility, absorption, secretion and blood flow. The functional repertoire of the ENS has recently been expanded by two independent reports showing that enteric neurons can also act as important immunoregulatory sentinels (Matteoli et al., 2014; Muller et al., 2014). Both studies demonstrate that enteric neurons show an intimate relationship with resident macrophages in the myenteric plexus and that both cell types communicate in a bidirectional manner. This concept was initially introduced in a murine model of postoperative ileus (POI), a condition characterized by impaired contractility of the intestine due to inflammation of the muscle layer (Matteoli et al., 2014). Electrical stimulation of the vagus nerve (VNS) improved functional outcome mainly through the reduction of muscular inflammation, an effect mediated by activation of cholinergic enteric neurons in close contact with resident macrophages of the myenteric plexus. Acetylcholine released by cholinergic neurons directly modulates macrophage responses via activation of the alpha7 nicotinic receptor (α7 nAChR) thereby dampening inflammatory responses in the muscle layer. These data convincingly demonstrate that the ENS controls intestinal immune responses via direct modulation of resident macrophages. Interestingly, Muller et al. (2014) recently reinforced the concept of neuron-macrophage interaction in the intestine and showed that resident macrophages and enteric neurons reciprocally interact under physiological conditions. Enteric neurons contribute to the maintenance of muscularis macrophages through the production of colony stimulating factor-1 (CSF-1), a growth factor necessary for macrophage survival. Consecutively, macrophages directly affect neuronal homeostasis via the release of bone morphogenic protein type 2 (BMP2), which binds to its receptor BMPRII on enteric neurons. The bidirectional interplay between muscularis macrophages and enteric neurons is essential to maintain proper tissue function under physiological conditions as selective depletion of muscularis macrophages led to disturbed peristaltic activity in the colon due to dysregulated contractions in the muscularis externa. The impaired contractility in the absence of muscularis macrophages was correlated with reduced neuronal activation of SMAD1, SMAD5 and SMAD8, the downstream signaling mediators of BMP receptors. Although the impact of disturbed BMP-signaling on enteric neuron homeostasis was not investigated, it is likely that the functional defects in the absence of muscularis macrophages are due to aberrant neuronal activity. Collectively, these novel findings provide remarkable insights into the reciprocal interaction between enteric neurons and resident macrophages and define neuroimmune crosstalk as a fundamental regulatory system of motility and immune responses in the gut wall.

Whether similar mechanisms are present in the intestinal mucosa remains thus far unknown. It is however well established that neural mechanisms contribute to the regulation of mucosal immune responses. Mice that were exposed to the mucosal irritant dextran sulfate sodium (DSS) after vagotomy showed increased susceptibility to develop colitis (Ghia et al., 2006). In line with this finding, electrical and pharmacological activation of the vagus nerve has emerged as a potential therapy for the treatment of inflammatory bowel disease. A recent study indeed showed that pharmacological activation of the vagus nerve reduced mucosal inflammation and decreased susceptibility to DSS- and dinitrobenzene sulfonic acid (DNBS) induced colitis (Ji et al., 2014). Although it was shown that these effects were established through cholinergic modulation of splenic immune cells, it cannot be ruled out that activation of the vagus nerve influences local immune responses in the intestinal mucosa, through activation of the ENS. The close proximity between mucosal immune cells, including macrophages, and enteric nerve fibers innervating the intestinal lamina propria indeed suggests that neural mechanisms may also influence mucosal immune responses (Gautron et al., 2013).

The intimate crosstalk between enteric neurons and intestinal macrophages generates new perspectives on the cellular and molecular players involved in neuroimmune interaction in the gut. These findings also suggest that neuroimmune crosstalk in the ENS and central nervous system (CNS) occurs through equivalent modes-of-action. Comparable to macrophages in the ENS, microglia in the CNS are under the continuous control of surrounding neurons and support neuronal homeostasis through secretion of neuroprotective factors such as brain derived neurotrophic factor (BDNF), nerve growth factor (NGF), vascular endothelial growth factor (VEGF) and insulin-like growth factor type 1 (IGF1; Garden and Möller, 2006; Chen and Trapp, 2015). In addition to this analogy, intestinal macrophages resemble microglia by their high expression of CX3CR1, a receptor that is implicated in neuron-microglia interaction (Limatola and Ransohoff, 2014; Paolicelli et al., 2014). In this perspective, shared and distinct features of microglia and intestinal macrophages as well as recent findings supporting the hypothesis that these two macrophage subsets are alike will be discussed below.

## Distinct and Common Features of Intestinal Macrophages and Microglia: from Phenotype to Function

Macrophages are common to every tissue, yet they display a high level of functional and phenotypical diversity (Wynn et al., 2013). This phenotypical heterogeneity enables tissue macrophages to carry out diverse and context-dependent tasks that meet the functional requirements of a specific tissue (Glass, 2015). Recent evidence suggests that macrophage phenotype is primarily determined by tissue-specific signals (Gordon et al., 2014). In this context one would intuitively assume that intestinal macrophages and microglia occupy two extremes of the phenotypical spectrum. Whereas intestinal macrophages reside in the least sterile environment of the body and are constantly exposed to foreign antigens, microglia are “born and raised” in an isolated, sterile environment with limited external influences (Prinz and Priller, 2014; Gross et al., 2015). Another distinction between intestinal macrophages and microglia is their ontogeny. Recent evidence shows that most tissue macrophage populations are derived from embryonic precursors from the yolk sac and/or fetal liver, whereas bone marrow monocytes do not contribute to the maintenance of tissue macrophages (Ginhoux and Jung, 2014). Intestinal macrophages seem to be the exception to this rule. Although yolk-sac and fetal liver derived macrophages are detected in the embryonic and early postnatal intestine, the intestinal macrophage pool in adult mice is continuously replaced by circulating blood monocytes (Bain et al., 2014). This feature is most likely dictated by the unique intestinal microenvironment, as influx of Ly6C^+^ CCR2^+^ monocytes largely depends on the presence of microbiota. In contrast to the distinct origin of intestinal macrophages, microglia are solely derived from yolk-sac progenitors that colonize the brain in early embryonic life and persist throughout adulthood (Kierdorf et al., 2013; Gomez Perdiguero et al., 2015; Hoeffel et al., 2015).

**Figure 1:**
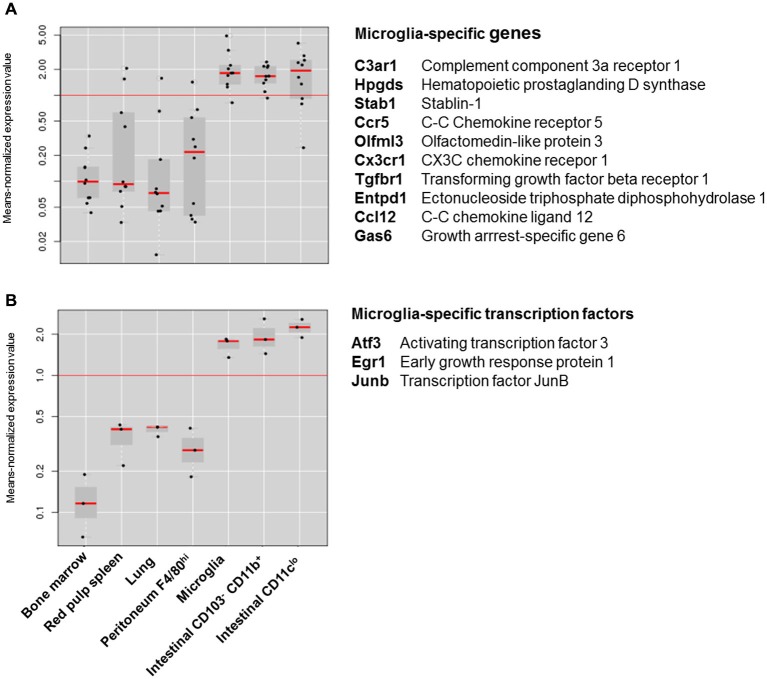
**Expression of microglia specific genes in different macrophage subsets. (A)** Box-and-whiskers plot of the means-normalized expression values of “microglia specific genes” *C3ar1, Hpgds, Stab1, Ccr5, Olfml3, Cx3cr1, Tgfbr1, Entpd1, Ccl12 and Gas6* in different macrophage subsets. **(B)** Box-and-whiskers plot of the means-normalized expression values of “microglia specific transcription factors” *Egr1, Atf3 and Junb* in different macrophage subsets. List of microglia specific genes is based on Butovsky et al. (2014); Gene expression of different macrophage subsets is based on publically available microarray data from Immunological genome consortium (ImmGen; Heng and Painter, 2008). Plots were obtained through the “My GeneSet databrowser” on the Immgen site. Long red horizontal line represents the mean expression of all values in the graph. Black dots are expression values of single genes relative to the mean expression. Gray boxes are box-and-whiskers plots with short red lines representing the median expression value within a macrophage subset.

Although these differences suggest that intestinal macrophages and microglia have few features in common, recent comparative transcriptomics on different macrophage subsets depict a different picture. In an attempt to identify a “macrophage core gene signature” that unifies different tissue macrophages, the Immunological Genome (ImmGen) Project showed that macrophage subsets display a high level of diversity and are merely unified by the expression of 39 genes (Gautier et al., 2012). Hierarchical clustering based on this 39-gene macrophage core signature showed that intestinal macrophages showed the strongest relationship with microglia. A similar observation was made in a recent study characterizing the molecular and functional signature of microglia (Butovsky et al., 2014). This study showed that microglia express a unique gene expression signature, characterized by the high expression of specific genes including *Cx3cr1*, *Fcrls, P2ry12, Tmem119, Olfml3, Hexb, Tgfbr1, Gpr34, Mertk* and *Gas6* that are not expressed by lung, spleen, peritoneal and bone marrow macrophages. However, unlike other tissue-resident macrophages, intestinal macrophages show high expression of several “microglia-specific” genes, including *Cx3cr1, Fcrls, P2ry12, Olfml3, Mertk and Gas6* (Figure 1A*)*. Moreover, the high expression of the transcription factors *Egr1, Atf3* and *Junb* in both macrophage subsets suggests that microglia and intestinal macrophages employ a similar transcriptional machinery to establish their phenotype (Figure 1B). In addition to their transcriptional resemblance, microglia and intestinal macrophages also have a significant overlap in functional phenotype. In steady state, both macrophage subtypes display a typical anti-inflammatory phenotype, characterized by high expression of tolerogenic markers. The tolerogenic features of mucosal macrophages are indispensable for the maintenance of intestinal immune homeostasis (Zigmond and Jung, 2013; Bain and Mowat, 2014a). Especially as the gastrointestinal tract is continuously exposed to vast amounts of foreign antigens, innocent microorganisms such as commensal bacteria or food antigens should be recognized as harmless to avoid unnecessary inflammation and collateral tissue damage. Macrophages residing in the intestinal mucosa are thus essential players in maintaining tolerance against harmless antigens. Instead of producing pro-inflammatory mediators in response to stimuli such as Toll-like receptor (TLR) ligand, mucosal macrophages produce high levels of IL-10 necessary for the maintenance of regulatory T cells (Smith et al., 2011; Pabst and Bernhardt, 2013). Also in the intestinal muscularis externa, resident macrophages acquire a tolerogenic phenotype in order to restrict the release of inflammatory mediators which can affect smooth muscle and neuronal function (de Jonge et al., 2005; Matteoli and Boeckxstaens, 2013). Similar mechanisms are also essential for normal CNS function as chronic release of inflammatory mediators can induce irreversible neuronal damage (Brown and Neher, 2010, 2014). How both microglia and intestinal macrophages are educated by their microenvironment to acquire a tolerogenic phenotype enabling them to perform context-dependent functions remains to be determined. However, recent research by the group of Glass convincingly showed that distinct tissue environment signals drive divergent gene expression programs in macrophages (Gosselin et al., 2014). Although macrophages share a common enhancer repertoire mainly driven by M-CSF or IL-34, tissue-specific signals induce the expression of divergent secondary transcription factors that collaborate with PU.1 to establish tissue-specific enhancers. Hence, based on the transcriptional and functional similarities between intestinal macrophages and microglia, it is reasonable to speculate that both macrophage subsets are conditioned by similar environmental factors.

## Macrophage Phenotype in the Intestine and Brain: a Matter of Innervation?

It is well established that the phenotypical and functional features of microglia in steady-state are mainly driven by neuronal signals. Healthy neurons in the CNS maintain microglia in a non-activated state via both secreted and membrane bound signals including CD200, CX3CL1, neurotransmitters and neurotrophins (Biber et al., 2007; Pocock and Kettenmann, 2007). One could argue that the “little brain of the gut” (i.e., ENS) functions as an important immunoregulatory system in the intestine, comparable to the situation in the “big brain” (i.e., CNS). It is furthermore highly intriguing that intestinal macrophages express high levels of CX3CR1, the sole receptor of CX3CL1 (Bain and Mowat, 2014b). The CX3CL1-CX3CR1 axis is one of the best described mechanisms of neuroimmune interaction in the CNS and has been implicated in many neurophysiological and neuropathological conditions. Its role in the control of inflammatory cytokine production by microglia has been proven to be therapeutically relevant in several models of neurodegenerative disorders including Alzheimer’s and Parkinson’s disease (Cardona et al., 2006; Lee et al., 2010). Interestingly, also in the intestine, CX3CL1-CX3CR1 signaling participates in the homeostatic control of inflammatory responses. CX3CR1-deficient mice not only show increased vulnerability to DSS induced colitis but also fail to develop oral tolerance to ovalbumin (Hadis et al., 2011; Medina-Contreras et al., 2011). The latter was correlated with reduced production of interleukin-10 by gut-resident macrophages affecting the proliferation of regulatory T cells. Accordingly, the CX3CL1-CX3CR1 axis is an important modulator of macrophage function in both the CNS and intestine and participates in the maintenance of a tolerogenic, anti-inflammatory environment in both tissues. Importantly, the high expression of CX3CR1 seems to be a unique feature of microglia and intestinal macrophages. As most other tissue-resident macrophages express low levels of this receptor, intestinal macrophages and microglia are likely exposed to similar tissue-specific factors driving CX3CR1 expression. Transforming growth factor β (TGFβ) has recently been identified as a factor produced by neurons and glial cells of the CNS, driving the expression of several microglia-specific genes, including CX3CR1 (Butovsky et al., 2014). Whether TGFβ is an important determinant of intestinal macrophage phenotype and is produced by neural cells in the ENS remains to be studied. Of note, different cell types of the intestinal mucosa, including epithelial cells and T cells, produce significant amounts of TGFβ (Feagins, 2010). Next to TGFβ, CX3CL1 could be an important modulator of intestinal macrophage function. Studies in CX3CL1-reporter mice have shown that intestinal epithelial cells and goblet cells are major producers of CX3CL1, most likely conditioning intestinal macrophages that are in close proximity to the lumen (Kim et al., 2011). A subset of mucosal macrophages residing in closer proximity to the submucosal plexus might be preferentially affected by ENS input, somewhat similar to the resident macrophages in the intestinal muscularis. Although this subset has not been described yet, several recent studies support the idea of a heterogeneous mononuclear phagocyte system in the intestinal mucosa based on the expression of certain surface markers. For example, mature CX3CR1^high^ macrophages can be subdivided in different subsets that either express high or low levels of CD11c. Interestingly, CD11c^low^ F4/80^+^ macrophages show a preferential localization at the bottom of the villi, in closer proximity to the submucosal plexus, whereas CD11c^high^ F4/80^+^ reside mainly at the subepithelial space (Rivollier et al., 2012; Koscso et al., 2015). Similarly, a novel CX3CR1^high^, CD11c^low^, CD169^+^ macrophage subset that was recently identified in the colonic mucosa also occupies an anatomical niche in close proximity to the muscularis mucosa (Asano et al., 2015). In contrast to the majority of colonic macrophages, this macrophage subset does not depend on microbiota-derived signals for its maintenance. Moreover, the functional features of this subset seem to deviate from those of subepithelial CX3CR1^high^ macrophages. Whereas subepithelial macrophages are refractory to pathogen-derived molecular signals, CD169^+^ mucosal macrophages produce significant amounts of inflammatory mediators upon epithelial injury serving as critical regulators of mucosal inflammation. This phenotypical and functional diversity suggests that the microenvironment in the intestinal mucosa harbors different systems involved in conditioning intestinal macrophages. Further scrutiny is required to demonstrate the existence of mucosal macrophages that resemble muscularis macrophages and can be conditioned by neuronal mechanisms.

## Conclusion

It is well established that the ENS strongly resembles the CNS, using a similar set of sensory, motor and inter neurons as well as a comparable set of neurotransmitters. The recent identification of neuron-macrophage crosstalk in the myenteric plexus suggests that the ENS and CNS also employ similar mechanisms of neuroimmune interaction. Although the gastrointestinal tract and the CNS are exposed to different environmental signals, it seems likely that both tissues produce equivalent conditioning factors that shape macrophage phenotype. Considering the transcriptional resemblance between microglia and intestinal macrophages, TGFβ is possibly an important determinant of macrophage phenotype in the intestine. Whether neural TGFβ is involved in this process remains to be determined, but it can be anticipated that different intestinal cell types cooperate to drive macrophage phenotype.

## Funding

This work was supported by funding from the European Research Council (ERC) Advanced Grant (ERC-2013-Adg): 340101 Cholstim.

## Conflict of Interest Statement

The authors declare that the research was conducted in the absence of any commercial or financial relationships that could be construed as a potential conflict of interest.
